# Severe Polyarticular gout mimicking sepsis after acute kidney injury: a case report

**DOI:** 10.1093/omcr/omag046

**Published:** 2026-04-28

**Authors:** Leon Schönfeld, Marko Stuhr, Lars Klinkert, Gritt Braunegger, Birte Diekmeyer, Bastian Schön, Nihal Wilde

**Affiliations:** Department of Internal Medicine IA, Bundeswehr Central Hospital, Rübenacher Straße 170, 56072 Koblenz, Germany; Department of Internal Medicine IA, Bundeswehr Central Hospital, Rübenacher Straße 170, 56072 Koblenz, Germany; Department of Internal Medicine IA, Bundeswehr Central Hospital, Rübenacher Straße 170, 56072 Koblenz, Germany; Department of Nuclear Medicine, Bundeswehr Central Hospital, Rübenacher Straße 170, 56072 Koblenz, Germany; Department of Nuclear Medicine, Bundeswehr Central Hospital, Rübenacher Straße 170, 56072 Koblenz, Germany; Department of Internal Medicine IA, Bundeswehr Central Hospital, Rübenacher Straße 170, 56072 Koblenz, Germany; Department of Internal Medicine IA, Bundeswehr Central Hospital, Rübenacher Straße 170, 56072 Koblenz, Germany

**Keywords:** gout, polyarticular arthritis, urate bullae, acute kidney injury, FDG-PET, sepsis mimic

## Abstract

Background: Severe gout flares may rarely present as systemic inflammatory syndromes, closely mimicking sepsis. Milk-of-urate bullae are an uncommon manifestation of extensive crystal burden and are typically described in chronic tophaceous gout. The combination of polyarticular involvement, extreme inflammatory markers, acute kidney injury (AKI), and bullous lesions represents a diagnostic challenge. Case Presentation: A 69-year-old man presented with generalized weakness and incapacitating migratory joint pain after lying on the floor for three days. Laboratory evaluation revealed acute prerenal kidney injury and a fulminant inflammatory response (CRP 47.7 mg/dl, leukocytes 16.8 × 10^3^/μl, procalcitonin 4.05 ng/ml), prompting a sepsis work-up and empiric broad-spectrum antibiotics. No infectious focus was identified. 18F-FDG PET-CT demonstrated intense tracer uptake in multiple finger and toe joints, particularly in both first metatarsophalangeal joints. Over subsequent days, the patient developed erythema, swelling, and painful limitation of these joints, followed by bullous skin lesions containing milky fluid. Serum uric acid was elevated at 9.61 mg/dl. Polyarticular gout was diagnosed. After partial renal recovery, anti-inflammatory therapy with naproxen and colchicine was initiated, resulting in rapid clinical improvement and spontaneous ulceration of the urate bullae. Inflammatory markers normalized. Urate-lowering therapy with allopurinol was started prior to discharge. Conclusion: This case illustrates how severe polyarticular gout can mimic fulminant sepsis with extreme inflammatory markers and FDG-PET hypermetabolism. Acute kidney injury acted as a trigger for catastrophic crystal inflammation. Milk-of-urate bullae reflected extensive systemic urate burden. Recognition of this pattern is essential to avoid diagnostic delay and unnecessary antimicrobial therapy.

## Introduction/background

Gout results from supersaturation of uric acid and deposition of monosodium urate (MSU) crystals in joints and soft tissues. Renal excretion is the primary elimination pathway, and impaired kidney function directly increases serum urate levels [1]. MSU crystals activate the NLPR3 inflammasome, leading to IL-1β–mediated neutrophilic inflammation and acute gout flares [2].

While gout typically presents as monoarthritis—classically involving the first metatarsophalangeal joint—polyarticular and systemic manifestations may occur in advanced disease [1]. Tophi are a hallmark of long-standing gout and affect approximately 10%–15% of patients. In rare cases, extensive crystal burden may lead to milk-of-urate bullae, an exceptionally uncommon manifestation that is usually associated with advanced tophaceous disease and only rarely observed during acute systemic flares without previously established tophi [1, 3, 4].

Extreme inflammatory responses resembling sepsis are unusual and diagnostically hazardous [5]. In such cases, gout may be misinterpreted as infection or malignancy, especially when imaging modalities such as FDG-PET demonstrate multifocal hypermetabolism [6, 7].

## Case description

A 69-year-old man was admitted with generalized weakness and diffuse, incapacitating joint pain. He reported having lain on the floor for three days due to pain but denied trauma, syncope, fever, weight loss, or night sweats. His history included arterial hypertension, previous humeral fractures, spondylodiscitis, and surgically treated gastric perforation. Medication consisted of acetylsalicylic acid and lercanidipine.

**Figure 1:**
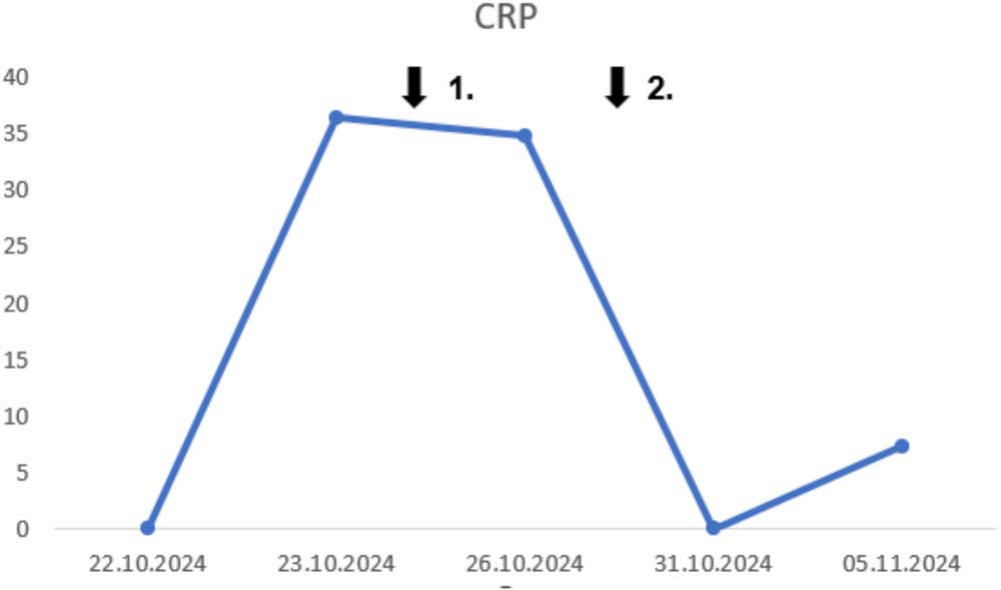
Temporal progression of C-reactive protein. Time point 1: Initiation of naproxen. Time point 2: Initiation of colchicine.

**Figure 2:**
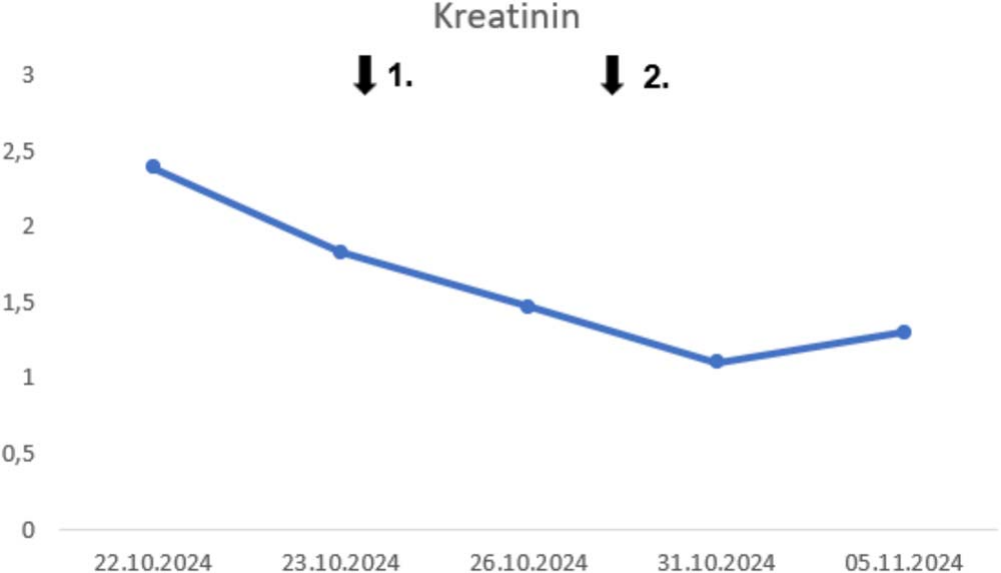
Temporal progression of creatinine. Time point 1: Initiation of naproxen. Time point 2: Initiation of colchicine.

On examination, joints were painful but showed full range of motion without overt inflammation. Initial laboratory testing revealed acute kidney injury (creatinine 2.39 mg/dl; BUN 108 mg/dl; eGFR 17 ml/min/1.73 m^2^) and an extreme inflammatory response (CRP 47.7 mg/dl, leukocytes 16.8 × 10^3^/μl, procalcitonin 4.05 ng/ml, Figures 1, 2).

Given the constellation of AKI and sepsis-like laboratory findings, an extensive infectious work-up was initiated. Chest CT, abdominal imaging, and blood cultures showed no infectious source. The inferior vena cava was collapsed on ultrasound, consistent with prerenal AKI due to dehydration. Empiric piperacillin–tazobactam was started.

**Figure 3:**
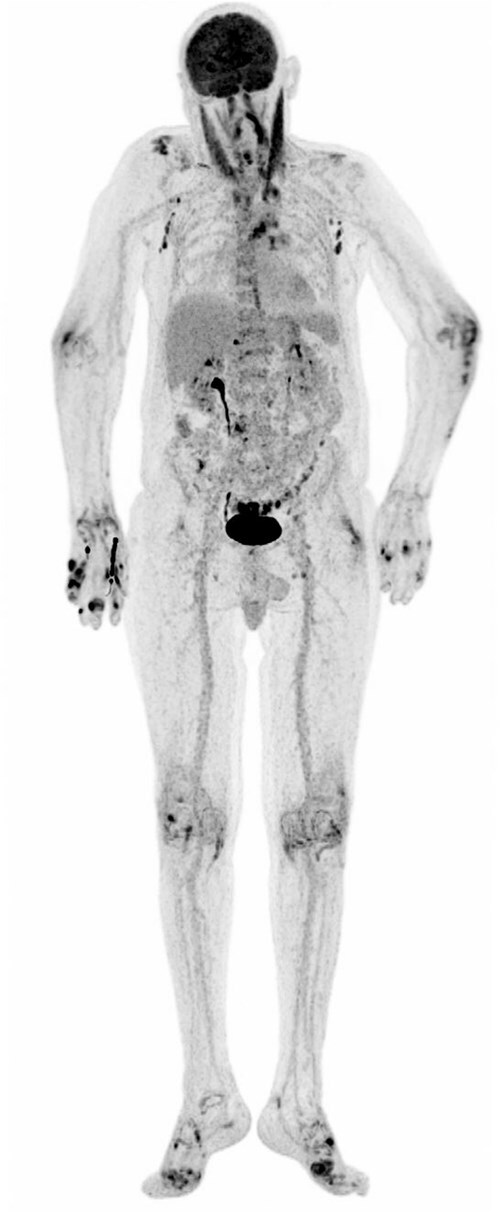
Maximum intensity Projecton (MIP) of18F-FDG PET showing distribution of focal joint hypermetabolism according inflammatory process especially in metacarpophalangeal and metatarsophalangeal joints also in bursas of knee, elbow and ACJ.

**Figure 4:**
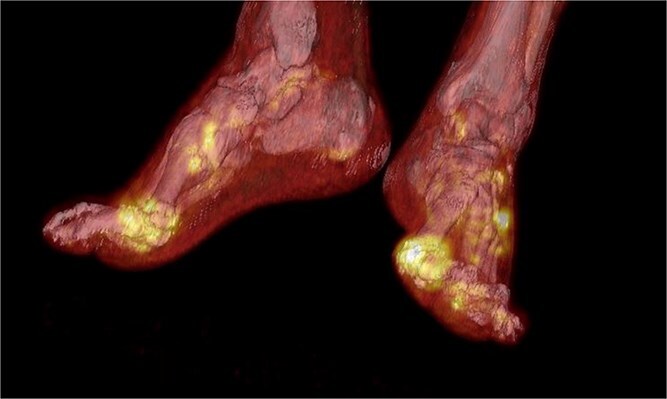
Fusion of 3D volume rendering and MIP of18F-FDG PET-CT demonstrate periarticular tissue swelling and hypermetabolism due to inflammation and arthritic bone errosions in first metatarsophalangeal joint bilaterally.

Despite fluid resuscitation and antibiotics, inflammatory markers remained markedly elevated and pain persisted. An 18F-FDG PET-CT was performed, revealing intense tracer uptake in multiple finger and toe joints, most prominently in both first metatarsophalangeal joints and the fifth digit of the right hand (Figures 3, 4).

**Figure 5:**
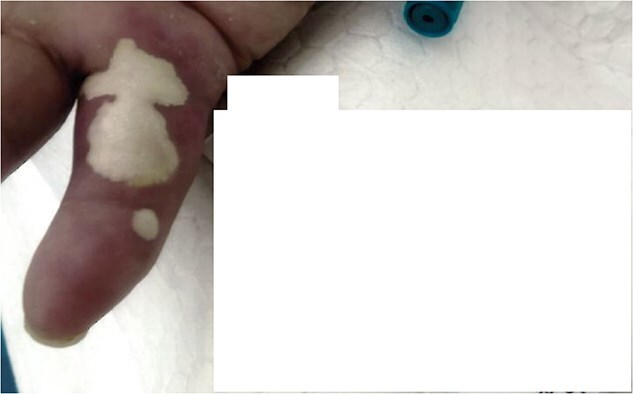
Digitus V with urate bulla.

Over subsequent days, the patient developed erythema, swelling, and restricted motion of these joints. Tense bullous lesions emerged on the affected toes and finger, containing milky fluid. Serum uric acid measured 9.61 mg/dl (Figure 5).

Polyarticular gout with urate bullae was diagnosed. After partial renal recovery, naproxen was initiated, followed by colchicine due to progression of symptoms. Rapid improvement ensued, with spontaneous ulceration of the bullae and marked pain relief. Inflammatory markers normalized. Allopurinol was commenced prior to discharge.

## Discussion

This case represents an exceptionally severe manifestation of gout, combining a sepsis-like systemic inflammatory presentation, markedly elevated procalcitonin, FDG-PET/CT hypermetabolism, and milk-of-urate bullae—features that are rarely reported together and pose a significant diagnostic challenge [8].

Severe gout flares are known to mimic systemic infection and may be clinically indistinguishable from bacterial disease. The markedly elevated procalcitonin observed in our patient is unusual for gout, as procalcitonin is typically low in crystal-induced arthritis, further reinforcing the sepsis-like presentation [5]. Renal dysfunction may have contributed to procalcitonin elevation and delayed its decline due to reduced clearance and altered inflammatory regulation in renal impairment; however, the markedly elevated level observed in our patient substantially exceeded proposed stage-adjusted thresholds for renal dysfunction, supporting a severe systemic inflammatory response beyond renal impairment alone [9, 10].

FDG-PET demonstrated multifocal hypermetabolism. Findings that are usually suggestive of infection or malignancy but have been repeatedly described in gouty arthritis and tophaceous disease [11].

Only the evolution of peripheral arthritis and bullous lesions redirected diagnostic reasoning toward crystal-induced disease. Acute prerenal kidney injury acted as the key trigger by abruptly increasing serum urate and precipitating widespread crystal inflammation [1].

Milk-of-urate bullae are rare and usually described in chronic tophaceous gout. Their presence reflects massive crystal burden and should be interpreted as a marker of systemic disease activity rather than a purely dermatologic phenomenon [3].

FDG-PET uptake in gout has been described in isolated reports and reflects metabolically active inflammation. This case underscores that FDG-PET positivity is not specific for infection and may represent crystal-induced immune activation.

Recognition of gout as a sepsis mimic is crucial to avoid prolonged antimicrobial therapy, unnecessary invasive diagnostics, and delayed anti-inflammatory treatment.

This case demonstrates that gout is not merely a localized crystal arthropathy but, in extreme presentations, a systemic inflammatory disease capable of imitating sepsis and malignancy.

Joint aspiration was not performed because the diagnosis became clinically evident based on the characteristic clinical course, FDG-PET/CT findings, and response to anti-inflammatory therapy, which represents a limitation of this case report.

**Learning Points**

- Demonstrates that severe gout can present as a *sepsis mimic* with extreme inflammatory markers—including markedly elevated procalcitonin—highlighting a critical diagnostic pitfall for internists managing patients with systemic inflammatory syndromes.
- Shows that 18F-FDG PET/CT hypermetabolism is not specific for infection or malignancy and may reflect crystal-induced immune activation, preventing misinterpretation of advanced imaging in complex diagnostic workups.
- Introduces milk-of-urate bullae as a visible marker of massive systemic crystal burden, reframing them from a dermatologic curiosity to a sign of advanced, systemically active gout requiring urgent anti-inflammatory intervention.
